# Safety and efficacy of telitacicept in refractory systemic lupus erythematosus patients who failed treatment with belimumab: A case series

**DOI:** 10.1007/s00393-023-01461-z

**Published:** 2023-12-29

**Authors:** Qiuyu Fan, Huiqin Yang, Ya Liu

**Affiliations:** Department of Rheumatology, No.1 Hospital of Wuhan, No. 215, Zhongshan Avenue, Wuhan, Hubei Province China

**Keywords:** Systemic lupus erythematosus, Telitacicept, Belimumab, Refractory, Hepatitis, Systemischer Lupus erythematosus, Telitacicept, Belimumab, Refraktär, Hepatitis

## Abstract

**Objective:**

This study aimed to determine the effect and safety of telitacicept, an antagonist of BLyS/APRIL-mediated B cell activation, in patients with systemic lupus erythematosus (SLE) who failed treatment with belimumab and in whom telitacicept was administered combined with conventional therapy. A review of published reports on telitacicept for SLE was also performed.

**Methods:**

A retrospective review was performed of the records of patients seen in the Department of Rheumatology at the Wuhan Hospital of Chinese and Western Medicine, Wuhan, China, with refractory SLE who had failed treatment with belimumab. The terms “systemic lupus erythematosus” and “telitacicept” were used to identify patients reported in the English medical literature.

**Results:**

Identified were 14 refractory SLE patients, 3 males (21%) and 11 females (79%). The median age was 32.9 years. The median disease duration was 8.9 years. Patients in this cohort received telitacicept for an average of 34.1 weeks (17–62 weeks) and the total SLE responder index 4 (SRI-4) response rate was 78.9% (*n* = 11). The mean SLE Disease Activity Index (SLEDAI) score declined from 8.6 at baseline (95% confidence interval [CI] 7.87–9.28) to 4.29 at the endpoint (95% CI 3.4–5.16). All cases (100%) had hypocomplementemia at baseline, and 7 cases (50%) reported normal C3 and C4 levels at the follow-up endpoint. At the observation endpoint, the 24‑h urinary protein value of the 13 cases with proteinuria (baseline 24‑h urinary protein > 0.5 g/d) displayed a reduction, and 3 values turned negative. Although some patients had low serum total immunoglobulin (Ig) levels, subnormal IgG levels, and absolute counts of peripheral blood lymphocytes after treatment, no serious infection was reported. One case was refractory lupus hepatitis confirmed by liver pathology, and upon change to change to telitacicept treatment, liver function returned to normal.

**Conclusion:**

This is the first case series in SLE patients who accepted telitacicept treatment after failed treatment with belimumab. Our case series and review of the literature show that telitacicept combined with the original standard treatment may significantly improve disease activity while reducing prednisone use. No major safety issues were seen in this group of patients. Telitacicept may be a promising drug for the treatment of refractory lupus hepatitis.

## Abstract (de)

**Ziel:**

Ziel der Studie war es, die Wirksamkeit und Sicherheit von Telitacicept, einem Antagonisten der BLyS/APRIL-vermittelten B‑Zell-Aktivierung, bei Patienten mit systemischem Lupus erythematosus (SLE) zu untersuchen, bei denen eine Therapie mit Belimumab versagt hatte und die Telitacicept in Kombination mit konventioneller Therapie erhalten hatten. Außerdem wurde eine Übersicht der Publikationen zu Telitacicept bei SLE erstellt.

**Methoden:**

Es wurde eine retrospektive Übersicht über die Daten der Patienten mit refraktärem SLE und Therapieversagen von Belimumab aus der Abteilung für Rheumatologie des Wuhan-Hospitals für chinesische und westliche Medizin, Wuhan, China, gegeben. Die Begriffe „systemischer Lupus erythematosus“ und „Telitacicept“ wurden verwendet, um Patienten zu finden, über die in der englischsprachigen medizinischen Literatur berichtet wurde.

**Ergebnisse:**

Dabei wurden 14 Patienten mit refraktärem SLE identifiziert, 3 Männer (21 %) und 11 Frauen (79 %). Das Durchschnittsalter betrug 32,9 Jahre. Die mediane Krankheitsdauer lag bei 8,9 Jahren. Die Patienten in dieser Kohorte erhielten Telitacicept für durchschnittlich 34,1 Wochen (17–62 Wochen), und die Gesamt-Responserate im SLE Responder Index 4 (SRI-4) betrug 78,9 % (*n* = 11). Der durchschnittliche Wert für den Systemic Lupus Erythematosus Disease Activity Index (SLEDAI) nahm von 8,6 zu Beginn (95 %-Konfidenzintervall [CI] 7,87–9,28) auf 4,3 am Ende der Studie ab (95 %-KI: 3,4–5,16). Bei sämtlichen Fällen (100 %) bestand eine Hypokomplementämie zu Beginn, und in 7 Fällen (50 %) wurden normale C3- und C4-Werte zum Ende des Follow-up angegeben. Zum Ende der Beobachtungsphase zeigte sich beim Proteingehalt im 24-h-Urin der 13 Fälle mit Proteinurie (Ausgangswert für Protein im 24-h-Urin > 0,5 g/Tag) eine Verminderung, und 3 Werte davon waren jetzt negativ. Obwohl einige Patienten niedrige Gesamtwerte für Immunglobulin (Ig) im Serum, subnormale IgG-Werte und absolute Lymphozytenzahlen im peripheren Blut nach Therapie aufwiesen, wurden keine ernstlichen Infektionen angegeben. In einem Fall bestand eine refraktäre Lupushepatitis, die durch die Leberpathologie bestätigt war, und beim Wechsel zur Telitaciceptbehandlung normalisierte sich die Leberfunktion wieder.

**Schlussfolgerung:**

Es handelt sich hier um die erste Fallserie von SLE-Patienten, die eine Telitaciceptbehandlung nach Therapieversagen mit Belimumab akzeptierten. Die vorliegende Fallserie und Literaturübersicht zeigen, dass Telitacicept in Kombination mit der ursprünglichen Standardtherapie zu einer signifikanten Verbesserung hinsichtlich der Krankheitsaktivität bei Reduzierung des Einsatzes von Prednison führen kann. Es wurden keine wesentlichen Sicherheitsprobleme in dieser Patientengruppe beobachtet. Telitacicept ist möglicherweise ein vielversprechendes Medikament für die Behandlung der refraktären Lupushepatitis.

## Introduction

Systemic lupus erythematosus (SLE) is a highly heterogeneous autoimmune disease characterized clinically by multiple system involvement and multiple positive autoantibodies, and symptoms that range from mild to severe and even life threatening [1]. The treatment drugs for SLE are constantly being updated and replaced, are not universally effective, and produce severe adverse effects [2]. Therefore, safe and effective new treatments for SLE are urgently needed.

At present, the pathogenesis of SLE is still unknown. Moreover, its clinical manifestations are highly heterogeneous, and no single mediator or approach can account for the complex pathogenesis. A number of studies have suggested that immune dysfunction at the level of B cells, T cells, cytokines, and macrophages is closely related to SLE pathogenesis [3]. B cells play a very important role in the generation of disease via disruption of immune balance and the production of a variety of autoantibodies, which provides a theoretical basis for the study of new therapeutic targets [4–7].

It is well known that levels of B lymphocyte stimulator (BlyS) and a proliferation-inducing ligand (APRIL) are elevated in patients with SLE. Telitacicept is a new kind of dual BLyS/APRIL inhibitor that effectively blocks proliferation of activated B lymphocytes. These results indicate that blockade of BLyS alone by belimumab may be worse than dual blockade by telitacicept. Telitacicept not only inhibits activated B cells, but also has the advantage of targeting long-lived plasma cells [8]. Based on past efficacy and safety data, telitacicept has been approved in China to treat patients with active SLE [4].

To date, there are no reported real-world data on application of telitacicept in SLE patients who have failed treatment with belimumab, either in China or in other countries. In this study, we aimed to explore the efficacy and safety of telitacicept in patients with SLE who failed belimumab treatment.

## Methods

The records of all patients diagnosed with SLE in the Department of Rheumatology at the Wuhan Hospital of Chinese and Western Medicine, Wuhan, China, from March 2022 to July 2023 were reviewed. The course of their illness until July 2023 or their last visit was noted. All patients fulfilled the American College of Rheumatology (ACR) criteria for SLE [9]. The extracted patient information was deidentified to ensure anonymity, and the study adhered to the tenets of the Declaration of Helsinki. Demographic data and age at administration of telitacicept in SLE patients were recorded. The use of immunosuppressive drugs or glucocorticoids, course of disease, and duration of follow-up after treatment with telitacicept were recorded.

All patients were administered telitacicept combined with standard treatment (glucocorticoids and/or immunosuppressants mycophenolate mofetil, cyclophosphamide, tacrolimus, cyclosporin, etc., and/or antimalarials). Telitacicept was subcutaneously injected once a week on the basis of standard treatment with 80 mg or 160 mg, and the choice of dosage was based on the severity of disease and financial situation of the patient. The SLE responder index 4 (SRI-4) was assessed monthly. Patients were enrolled at different starting points, but all were observed at the same endpoint (July 01, 2023).

## Results

### Case series of patients

A total of 14 patients had used telitacicept after failing treatment with belimumab combined with conventional therapy. The demographic data, clinical manifestations, and treatment at the time of the first telitacicept administration are shown in Table 1. The endpoint of follow-up for all cases was 17–62 weeks. Among the 14 cases of SLE, three were males (21%) and 11 were females (79%). Median age was 32.9 years. The median duration of disease was 8.9 years. All cases were refractory SLE, among which 12 cases were patients with lupus nephritis. Prior to injection of telitacicept, 5 patients had received ≥ 2 immunosuppressants, and all patients had terminated belimumab treatment at least 6 months prior. These immunosuppressive drugs included mycophenolate mofetil (*n* = 7), azathioprine (*n* = 1), calcineurin inhibitors (*n* = 8), and leflunomide (*n* = 2). The median length of the disease course was 8.9 (1–18) years, and the median Systemic Lupus Erythematosus Disease Activity Index (SLEDAI) score was 8.6 (8–12) points. Among all 14 patients, the decision to inject telitacicept was due to persistent and recurrent conditions and difficulty in reducing glucocorticoids. Patients received medication at a dose of 160 mg (*n* = 7) or 80 mg (*n* = 7) every week. The specific values of 24‑h urine protein before and after treatment with belimumab are shown in Table 1.

**Table 1:** The baseline conditions of systemic lupus erythematosus patients before treatment with telitacicept

Case	Age (years)	Gender	Disease duration (years)	Belimumab (10 mg/kg), number of times	Before belimumab, 24‑h urinary protein (mg/24 h)	After belimumab, 24‑h urinary protein (mg/24 h)	Low complement level	Prior to telitacicept treatment	
								Glucocorticoids(mg)	Immunosuppressant
1	24	Female	6	8	300	350	Yes	14^a^	Aza 50 mg bid + HCQ
2	26	Male	14	8	3600	2027.2	Yes	10^a^	MMF + FK + HCQ
3	33	Male	6	8	3675	4409	Yes	20^b^	MMF + FK
4	26	Female	3	10	1208	1085.4	Yes	17.5^b^	FK + HCQ
5	28	Female	6	9	1807	1534.5	Yes	15^b^	MMF + HCQ
6	45	Female	12	8	1890	1550	Yes	20^b^	MMF + HCQ
7	35	Female	5	8	2306	2202.5	Yes	10^a^	LEF + FK
8	16	Female	9	11	1200	1025.6	Yes	15^b^	CsA
9	39	Female	9	8	4008.6	4985.5	Yes	20^b^	FK + HCQ
10	19	Male	1	8	1560	1061	Yes	14^a^	MMF + HCQ
11	46	Female	9	13	1205.8	896	Yes	8^a^	HCQ + HCQ
12	49	Female	18	8	1823.8	1500	Yes	20^b^	LEF + CsA + HCQ
13	32	Female	10	10	8345.6	9560.5	Yes	17.5^b^	MMF + FK + HCQ
14	43	Female	17	10	4367.2	5455.9	Yes	20^b^	MMF

*LEF* leflunomide, *MMF* mycophenolate mofetil, *CsA* cyclosporine A, *FK* tacrolimus, *Aza* azathioprine, *HCQ* hydroxychloroquine

^a^methylprednisolone

^b^prednisone acetate

### Primary outcome

Telitacicept was given for at least 17 weeks in all 14 cases. At the endpoint of observation, patients in the cohort had received telitacicept for an average of 34.1 weeks (17–62 weeks), and the total SRI‑4 response rate was 78.9% (*n* = 11). The response rate was 100% (3/3) in the 160-mg group. The mean SLEDAI score declined from 8.6 at baseline (95% confidence interval [CI] 7.9–9.3) to 4.3 at the endpoint (95% CI 3.4–5.2). In terms of the British Isles Lupus Assessment Group (BILAG) index, 14 cases (100%) achieved no additional organ reaching BILAG grade A or no more than one additional organ elevating to grade B. The Physician Global Assessment (PGA) did not increase ≥ 0.3 in any of the 14 patients (100%), indicating either stable or improving with no deterioration (Table 2).

**Table 2:** Primary outcome of telitacicept treatment in systemic lupus erythematosus patients

		SRI‑4	SLEDAI score		BILAG1	PGA score	
Case	Dose (mg), frequency, number of weeks	Endpoint *n* = 14	Pretreatment *n* = 14	Posttreatment *n* = 14	Posttreatment *n* = 14	Pretreatment *n* = 14	Posttreatment *n* = 14
1	160, qw × 62	Response	8	2	YES	1.5	0.5
2	160, qw × 61	Response	8	4	YES	1.2	0.8
3	160, qw × 55	Response	8	2	YES	1.5	0.8
4	160, qw × 43	Response	12	4	YES	1.5	0.5
5	160, qw × 31	Response	8	4	YES	1.5	0.8
6	160, qw × 39	Response	8	4	YES	1.2	0.8
7	160, qw × 35	Response	10	4	YES	1.2	0.8
8	80, qw × 31	No response	8	8	YES	1	0.8
9	80, qw × 27	No response	8	6	YES	1	0.8
10	80, qw × 23	Response	8	4	YES	1	0.8
11	80, qw × 19	No-response	8	6	YES	1.5	1.2
12	80, qw × 18	Response	10	4	YES	1.5	0.5
13	80, qw × 17	Response	8	4	YES	1.5	0.8
14	80, qw × 17	Response	8	4	YES	1	0.8

*SLEDAI *Systemic Lupus Erythematosus Disease Activity Index, *BILAG *British Isles Lupus Assessment Group *PGA *Physician Global Assessment, *qw *weekly

### Secondary outcome

**Fig. 1:**
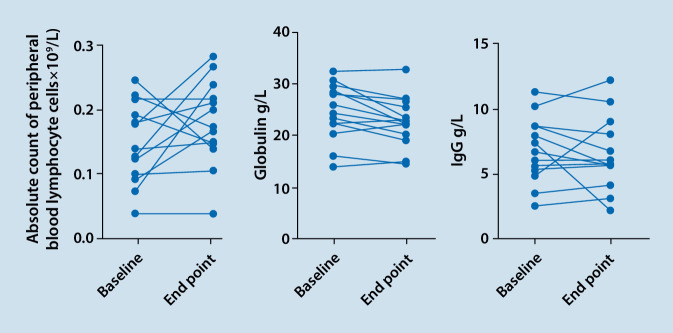
Secondary outcome of telitacicept in systemic lupus erythematosus

Twelve of the remaining 14 cases achieved a reduction in glucocorticoid administration by > 25% or a maintenance dose of ≤ 7.5 mg daily, of which 4 cases maintained a prednisolone dose ≤ 7.5 mg/d, and no cases increased glucocorticoid dosage. Fourteen cases had both C3 and C4 reexamined, and all cases showed either a stable or upwards trend; among them, all cases (100%) had hypocomplementemia at baseline, and 7 cases (50%) reported normal C3 and C4 levels at the end of follow-up. At the observation endpoint, the median 24‑h urinary protein value of the 13 cases with proteinuria (baseline 24‑h urinary protein > 0.5 g/d) showed a reduction, and 3 of them became negative. Among the 14 cases with hypoalbuminemia, plasma albumin increased to normal levels in four cases, and one case was not reexamined. Seven cases were subjected to reexamination of the anti-dsDNA antibody titer at the endpoint, and the antibody had become negative in 3 of these cases (Table 3). Between baseline and the endpoint, serum total immunoglobulin decreased in 9 patients, serum IgG decreased in 4 patients, and peripheral blood lymphocytes decreased in 6 patients, among whom 2 patients had a serum IgG level < 4.5 g/L, and there was no statistical difference in the above indicators (*p* = 0.403, *p* = 0.148, *p* = 0.285; Fig. 1). No serious infection was reported. Case 1 was a refractory patient with lupus hepatitis confirmed by liver pathology. Belimumab combined with the standard treatment was ineffective for 6 months, but after changing to telitacicept treatment, the liver function returned to normal.

**Table 3:** Secondary outcome of telitacicept in systemic lupus erythematosus

Case	Glucocorticoid reduction		Decreased anti-ds DNA antibody titer^c^	C3 (pre/post; g/L)	C4 (pre/post; g/L)	24‑h urinary protein (mg/24 h)	Plasma albumin (change trend)
	Before (mg)	After (mg)					
1	14^a^	6^a^	Negative	Low/N	N/N	N	Rise
2	10^a^	10^a^	Negative	Low/N	N/N	N	Rise^e^
3	20^b^	7.5^b^	Negative	Low/N	Low/N	Low	Rise
4	17.5^b^	7.5^b^	Yes	Low/rise	Low/low	N	Rise^e^
5	15^b^	10^b^	Yes^d^	Low/N	N/N	Low	Rise
6	20^b^	10^b^	Negative	Low/rise	N/N	Low	Rise
7	10^a^	8^a^	Negative	Low/rise	Low/N	Low	Rise
8	15^b^	10^b^	No	Low/low	N/N	Low	Descend
9	20^b^	10^b^	Negative	Low/rise	N/N	Low	Rise
10	14^a^	4^a^	No	Low/rise	N/N	N	Rise^e^
11	8^a^	6^a^	No	Low/N	N/N	Low	Rise
12	20^b^	10^b^	Yes^d^	Low/N	N/N	Low	Rise^e^
13	17.5^b^	10^b^	Yes^d^	Low/rise	N/N	Low	Rise
14	20^b^	10^b^	Negative	Low/N	N/N	Low	Rise

*N* normal

^a^methylprednisolone

^b^prednisone acetate

^c^the anti-dsDNA antibody changes from positive to negative, or the titer of anti-dsDNA antibody decreases compared with that of the baseline

^d^anti-dsDNA antibody turned negative

^e^plasma albumin turned negative

### Drug discontinuation and adverse events

All patients were still on treatment at the end of follow-up. One case of urinary tract infection and one case of pneumonia were reported. Both were classified as moderate, and both recovered after systemic treatment. No deaths were reported.

## Discussion

Systemic lupus erythematosus is a chronic inflammatory relapsing autoimmune disease with multiple clinical manifestations and positive autoantibodies [12]. Patients with SLE suffer from chronic organ injury due to both active disease and multidrug toxicity [10]. While the prognosis of SLE has greatly improved over the past 40 years, there is still an urgent need for a safer and more effective treatment, particularly in patients with recurrent disease or difficulty with glucocorticoid reduction [14].

Belimumab, an anti-BAFF monoclonal antibody, was approved by the United States Food and Drug Administration (FDA) for use in treating SLE. Belimumab has recently been widely used for treatment of SLE involving various organs [15].

Telitacicept (tradename: Tai’ai) is a fusion protein comprising a recombinant transmembrane activator and calcium modulator and cyclophilin ligand interactor (TACI) receptor fused to the fragment crystallizable (Fc) domain of human immunoglobulin G (IgG). Atacicept is also a soluble, fully human, recombinant fusion protein that inhibits the B cell-stimulating factors APRIL and BLyS and reduces B lymphocyte numbers and immunoglobulin levels in some studies. However, one study was terminated after enrollment of 6 patients due to an unexpected decline in serum IgG and occurrence of serious infections [13]. In the current study, some patients had decreased IgG levels after treatment, and IgM levels also slightly decreased; no cases of severe infection occurred. The level of immunoglobulin may be a reliable indicator for assessing the level of immunosuppression and adjusting the telitacicept dose, and further research is needed.

It was discovered in the current study that in the 14 patients with refractory SLE, disease activity could be relieved or controlled within a short period of time after administration of telitacicept. The drug retention rate of the 14 SLE cases was 100% during the observation period. There was no self-discontinuation caused by difficulties in administration or adverse reactions. A real-life observational study of 20 cases of SLE showed that telitacicept is a potential treatment option for patients with SLE, especially for lupus nephritis [13]. Consistent with the above report, in the present study, 13 of 14 cases had experienced repeated albuminuria before accepting telitacicept treatment, and urinary protein was relieved to varying degrees in 12 patients. As mentioned previously, reducing the glucocorticoid dose is a key treatment goal in SLE [11]. Similar to other literature reports [14, 15], a key finding of this study was the reduction in corticosteroids and no cases of upregulated glucocorticoid dosages. From case 1, it was found that telitacicept may be a promising drug for treatment of refractory lupus hepatitis, but this still needs to be confirmed in a large-sample study.

In contrast to other studies, telitacicept was well tolerated: adverse events were mild to moderate, and all resolved after symptomatic treatment, with one case of urinary tract infection and one case of pneumonia reported [16]. The study has some limitations. First, in this observational study of SLE, the number of subjects was small. The second possible bias of this study is the relatively short median observation time, which was only 7–53 weeks. The third possible bias is that belimumab may have enhanced the effect of telitacicept. In this study, all patients terminated treatment with belimumab and started using telitacicept 5 weeks later, and a pharmacokinetic study on belimumab showed that in the intravenous group, the mean serum belimumab concentration progressively decreased to 15.7% at 672 h (day 29) and to 3% at 1680 h (day 71) [17]. The estimated population terminal half-life was 19.4 days. Therefore, after 5 weeks there was very little residual belimumab, which likely did not have an impact on the subsequent evaluation of the efficacy of telitacicept, although further research is needed. Despite these limitations, the study provides valuable information about the effect of telitacicept treatment in patients with active autoantibody-positive SLE. Despite these limitations, to the best of the authors’ knowledge, this is the first observational study in patients with SLE who have failed treatment with belimumab.

In conclusion, this study found that telitacicept is a potential treatment option for patients with SLE, especially in lupus nephritis, with a significantly increased SRI‑4 response rate and reduced glucocorticoid use.

## Declarations

All procedures performed in studies involving human participants were in accordance with the ethical standards of the institutional and/or national research committee and with the 1964 Helsinki declaration and its later amendments or comparable ethical standards. Informed consent was obtained from all individual participants included in the study.
